# The effects of rapamycin on lens epithelial cell proliferation, migration, and matrix formation: An in vitro study

**Published:** 2010-08-16

**Authors:** Hongling Liu, Guangzhong Feng, Lan Wu, Shaoying Fu, Ping Liu, Wei Yang, Xiaomei Zhang

**Affiliations:** 1 Department of Ophthalmology, The 1st Affiliated Hospital, Harbin Medical University, Harbin, P.R. China; 2 Daqing Field Genaral Hospithal, Daqing, Heilongjiang, P.R. China; 3 Department of Cardiology, The 1st Affiliated Hospital, Harbin Medical University, Harbin, P.R. China

## Abstract

**Purpose:**

The objective of the present study was to investigate the efficacy of rapamycin on rabbit lens epithelial cell proliferation, migration, and secrcetion of extracellular matrix fibronectin (Fn).

**Methods:**

Rabbit lens epithelium cells (rLECs) were isolated from 1 month old rabbit. rLECs were either cultured for 24, 48, or 72 h with different doses of rapamycin (0.1, 1, and 10 ng/ml). The proliferation kinetics, proliferating cell nuclear antigen (PCNA) expression, and basic fibroblast growth factor (bFGF)-induced migration of rLEC was determined by methyl thiazol tetrazolium (MTT) assay, western blotting and transwell chamber assay, respectively. The effect of rapamycin on the synthesis of Fn was examined via immunofluorescence.

**Results:**

Rapamycin significantly inhibited rLEC proliferation and PCNA protein expression when administered doses and time periods except for 0.1 ng/ml for 24 h. bFGF-induced migration rLECs was inhibited by pretreatment with rapamycin for 48 h. Extracellular matrix Fn formation of rLECs was also reduced by rapamycin.

**Conclusions:**

In our study, rapamycin strongly inhibited rLEC proliferation, bFGF-induced migration, and extracellular matrix Fn formation. Thus, rapamycin may have a potential inhibition of posterior capsule opacificatin (PCO) and needs further study.

## Introduction

Posterior capsule opacification (PCO), known as after-cataract, is the main long-term complication of extracapsular cataract extraction (ECCE), with an estimated incidence of 20%–40% of cases within 5 years after surgery [1]. The cellular mechanisms responsible for PCO are still unclear. Previous studies have suggested that the proliferation, migration, and epithelial-mesenchymal transition (EMT) of the remaining lens epithelial cells (LECs) after cataract surgery are a major cause of PCO [2]. The response of LECs can be considered a wound-healing reaction resulting from the activation of inflammatory cells and production of cytokines and growth factors after surgery, influenced by the extracellular matrix (ECM) of the lens capsule [3,4].

The inhibition of LECs proliferation, migration and secreting extracellular matrix would be an ideal way to prevent this complication. Rapamycin is one kind of potent immunosuppressant and antiproliferative drug. Rapamycin-eluting coronary stent has been safely and clinically used, which remarkably reduced the rates of restenosis and associated clinical events after percutaneous coronary revascularization. The mechanisms may be by inhibiting proliferation and migration of smooth muscle cells (SMCs) [5-7].

In our previous study, RAPA was loaded into the polylactide-glycoli acid (PLGA) layer on the surface of intraocular lens (IOLs) which prevented formation and development of PCO in rabbit model for 24 weeks [8]. The topical application of immunosuppressive drug with anti-inflammatory and antiproliferative effects seems to be promising. However, there is still a lack of information about the biologic effects of rapamycin on lens epithelium cells. Thus, the aim of this study was to evaluate the effects of rapamycin on the three major processes of PCO (proliferation, migration, and matrix synthesis capability) of rabbit lens epithelium cells (rLECs) in vitro.

## Methods

### Cell culture and treatments

All procedures were conducted in accordance with institutional guidelines for the use of animals in scientific research and adhered to the ARVO Statement for Use of Animals in Ophthalmic and Vision Research. rLECs were isolated from the lenses of White New Zealand Rabbits at 1 month of age. The whole eyes were rinsed in 96% ethanol for 30 s to minimize a possible bacterial contamination of the lenses from the eye surface during lens preparation and followed by washed in phosphate buffered-saline solution (PBS). The cornea was dissected aseptically and the lens was released using a sterile plastic tube. Any remainders of zonular fibers were cut off. The whole lenses (20 lenses) were incubated in 30 ml of Dulbecco’s modified Eagle’s Minimum Essential Medium (D-MEM; Sigma, St Louis, Mo) supplemented with 10% fetal bovine serum (FBS, Pan-systems, Bochum, Germany). The anterior capsule membranes including equator region were collected by curvilinear continuous capsulorrhexis (CCCE). After cutting with fine scissors, the anterior capsule membranes (1 mm^2^) with attached epithelial cells were centrifuged (112× g, 5 min), resuspended in 1 ml of 10% FBS, and transferred to a 35 mm culture dish in DMEM containing 10% FBS, 100 U/ml penicillin G, 100 μg/ml streptomycin, and 2.5 μg/ml amphotericin-B. The lens capsular explants were incubated undisturbed for 7 days in a humidified atmosphere of 5% CO_2_ at 37 °C. Upon becoming confluent in 2 weeks, the proliferating rLECs were trypsinized and subcultured in DMEM containing 10% FBS. Early-passage of rLECs was defined as subcultured cells in media containing 10% FBS until 1–3 passages. The passage 3 cells were seeded with a density of 50,000 cells in 96-well plates for overnight culture. Culture medium was changed and rapamycin (Sigma) was added in different doses. Culture media of all groups were maintained in duplicates and were incubated for 24, 48, or 72 h. After completion of the incubation periods, rLEC were used to observe morphological changes after hematoxylin and eosin (HE) staining, methylthiazol tetrazolium (MTT) assay, transwell chamber assay, immunofluorescence assay, and western blotting.

### MTT assay

Cell proliferation was assessed by the MTT assay [9]. After the cells were treated with different doses (0, 0.1, 1, 10 ng/ml) rapamycin for 24, 48, or 72 h, 10 μl of 5 mg/ml MTT (in PBS) was added to each well of a 96-well plate, and continually incubated 4 h at 37 °C. The formazan granules obtained in cells were then dissolved in dimethyl sulfoxide (DMSO). The absorbance values were detected at wavelength of 570 nm by a 96-well multiscanner autoreader (MR 5000; Dynatech, Chantilly, VA). The experiments were performed 4 times. Cell reduction of MTT (%)=(1 -OD of treated cells/OD of control cells) ×100%.

### Transwell chamber assay

To evaluate the effects of rapamycin on the migratory ability of rLECs, rLECs were plated at a density of 60,000/ml in a 25-cm^2^ flask. After 2 days, the medium was changed, and rapamycin was added at doses of 0, 0.1, 1, and 10 ng/ml. After treatment for 48 h, cells were harvested by trypsinization. Cells were then seeded at a density of 100,000 cells in 0.2 ml in the upper compartment of a 2-chamber migratory well (Costar, 8-μm pore size). In the lower compartment 0.8 ml of medium was supplemented with 3 ng/ml basic fibroblast growth factor (bFGF). After incubation for 24 h, cells were removed from the upper side of the membrane inset, the upper cell layer was removed with a cotton swab, and the cells on the lower side were fixed with 4% formaldehyde solution. Subsequently, cells were stained with crystal viola and counted under a microscope.

### Immunofluorescence assay

To evaluate the capability of the cultured rLECs to synthesize extracellular matrix fibronectin (Fn) and proliferating cell nuclear antigen (PCNA), rLECs were incubated with rapamycin at doses of 0, 0.1, 1, and 10 ng/ml for 24 h in 6-well plates. The cells were fixed and analyzed by immune fluorescence. In detail, after incubation for 24 h, the culture medium was removed. Cells were washed twice with PBS and fixed with ice-cold methanol for 10 min at –20 °C. Cells were washed twice with PBS. Ten microliters of the first antibody against Fn (Millipore, Billerica, MA) and PCNA (Millipore) were added for 30 min at 37 °C. Cells were washed twice with PBS. One hundred microliters of the second antibody conjugated to fluorescein isothiocyanate (RAM-FITC, Zhongshan Goldenbridge, Beijing, China) was added for 1 h at 37 °C. Cells were washed twice with PBS. Immunofluorescence was detected by a Nikon E800 microscope with epifluorescence (Nikon, Tokyo, Japan). Each image was photographed with a digital camera for the same exposure time.

### Western immunoblot

To evaluate the effects of rapamycin on synthesize Fn and PCNA protein expresion, rLECs were incubated with rapamycin at doses mentioned above for 24 or 48 h. Cells were washed in PBS three times, scraped with cell scraper, centrifuged (10,303× g, 6 min). The cells were homogenized with protein extraction reagent (Solarbio Science Technology Co, Beijing, China) containing protein lysate (100 μl),proteinase inhibitors and PMSF (2 μl). The protein concentrations were quantitated by the bicinchoninic acid assay (Sigma-Aldrich, St. Louis, MO). The samples containing 30–50 μg protein were separated on 12% SDS-polyacrylamide gels and electrophoretically transferred to nitrocellulose membranes (Bio-Rad, Hercules, CA). The membranes were blocked at room temperature for 1 h in TBS-T (10 mM Tris-HCl [pH 7.6], 150 mM NaCl, 0.05% Tween-20) containing 5% nonfat dry milk and incubated overnight at 4 °C with anti-PCNA (Millipore), β-actin (Boster, Wuhan, China) or Fn antibodies (Millipore). The blots were then incubated for 1 h at room temperature with horseradish peroxidase conjugated secondary antibodies (Zhongshan Goldenbridge, Beijing, China), and bands in membranes were detected using enhanced chemiluminescence reagent on autoradiographic film.

### Statistical analysis

Results are expressed as the mean±SD. Statistical significance was determined by the two-tailed student’s *t*-test and differences at p0>0.05 were considered as statistically significant.

## Results

### Effects of rapamycin on rLECs proliferation

**Figure 1:**
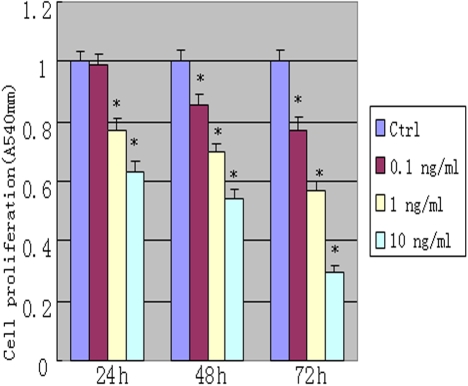
Inhibitory effect of rapamycin on proliferation in rabbit lens epithelium cells (rLEC). Cells were treated with different doses of rapamycin for 24 h, 48 h, 72 h. The cell proliferation was assessed by methylthiazol tetrazolium (MTT) assay. Relative proliferation(%)=(OD of treated cells/OD of control cells)×100. *p<0.05, compared with the control cells.

**Figure 2:**
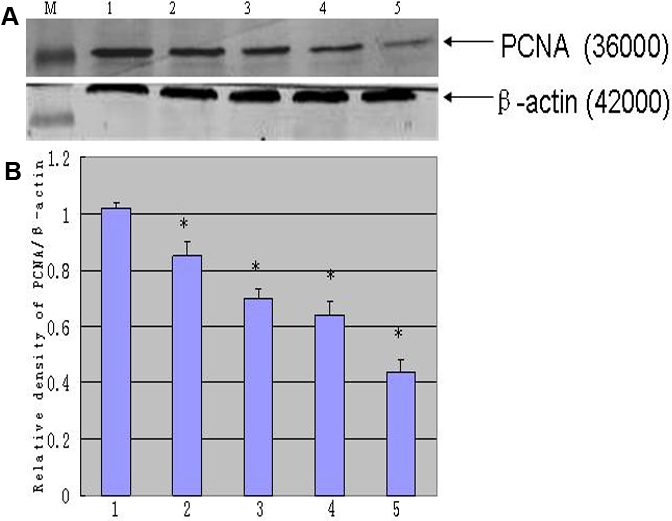
Western blotting was detected of proliferating cell nuclear antigen (PCNA) in rLEC. PCNA, and β-actin were determined in rLECs by western blotting (**A**). The blot was stripped reprobed with anti-β-actin as an internal control: Lanes M, 1, 2, 3, 4 and 5 are protein marker, normal control, 0.1 ng/ml for 24 h, 0.1 ng/ml rapamycin for 48 h, 10 ng/ml rapamycin for 24 h, and 10 ng/ml rapamycin for 48h, respectively. The histogram (**B**) summarizes the results of scanning densitometry of PCNA in the blot. Data were standardized by an internal loading control (β-actin). All differences were statistically significant, *p≤0.05.

**Figure 3:**
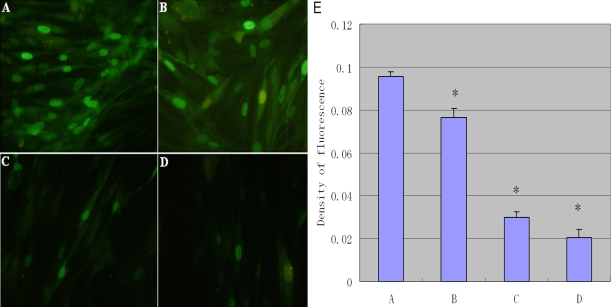
Immumofluorescence observation (100×) of PCNA (oval green spots) in rLEC treated with different doses of rapamycin. Panel **A** shows the control rLECs, while **B**, **C**, and **D** shows 0.1, 1, 10 ng/ml rapamycin, respectively, after a 24 h exposure. PCNA is clearly reduced in the rapamycin-treated group. The histogram (**E**) summarizes the results of scanning densitometry of PCNA. All differences were statistically significant, *p≤0.05.

**Figure 4:**
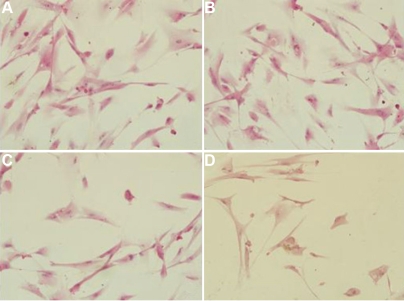
The cell morphological observation (100×) after hematoxylin and eosin (H&E) staining. Panel **A** shows the control rLECs, while **B**, **C**, and **D** shows 0.1, 1, 10 ng/ml rapamycin, respectively, after a 48 h exposure. Rapamycin had no effect on rLECs morphology treated with different doses.

Active mitochondria of living cells can cleave MTT to produce a purple-blue formazan, the amount of which gives an indication of the number of the living cells. We examined the effect of rapamycin on proliferation of rLECs. Although the lower concentration (0.1 ng/ml) of rapamycin has no effect on cell proliferation within 24 h incubation, rapamycin significantly inhibited proliferation of rLEC in a dose-dependent manner. The inhibitory rates were 36% and 23% at doses of 1.0 and 10 ng/ml rapamycin, respectively. Rapamycin treatment also inhibited rLEC proliferation with relative low concentration (Figure 1). rLECs treated with 1.0 ng/ml of rapamycin, proliferations of rLECs at 24, 48 and 72 h were decreased by 22.8%, 29.9% and 43.2%, respectively, when compared to the control cell. In addition, rLECs treated with 10 ng/ml of rapamycin at 24, 48 and 72 h, the inhibitory rates were significantly inhibited by 36.5%, 46.2% and 70.3%, respectively, when compared to the control cell. These findings were also confirmed by the observed expression of PCNA protein, a marker for cell proliferation (Figure 2 and Figure 3). Rapamycin also significantly decreased (p<0.05) PCNA protein expression in a dose- and time dependent manner. Additionally, no difference in morphology was observed in rLECs among rapamycin-treated and untreated cultures (Figure 4).

### Effects of rapamycin on rLECs migration

**Figure 5:**
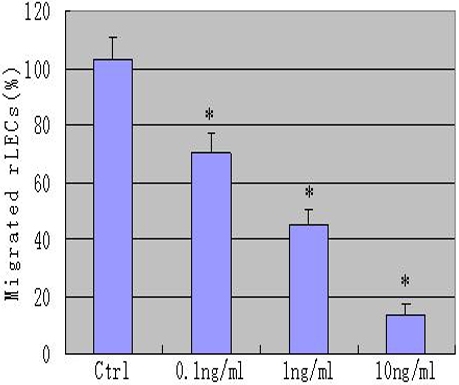
rLECs migration observation. The x-axis represents the experimental groups and the y-axis for the number of migrated cells per membrane segment. Basic fibroblast growth factor (bFGF)-stimulated cell migration was decreased with increasing doses of rapamycin. There was only 14% migratory activity at 10 ng/ml, approximately 45% at 1 ng/ml and 70% at 0.1 ng/ml rapamycin groups when compared to the control cells. The values are given in mean±SD. Based upon the Tukey-Kramer test for multiple comparisons, all groups significantly differed from each other (*p≤0.05).

bFGF-stimulated rLECs caused cell migration. Rapamycin evidently decreased bFGF-stimulated rLEC migration. The migratory activities were only 14%, 45%, and 70% at 10, 1 and 0.1 ng/ml rapamycin, respectively, when compared to the control cells (Figure 5). All groups were statistically different from each other (p<0.05).

### Expression of Fn produced by rLECs in vitro

**Figure 6:**
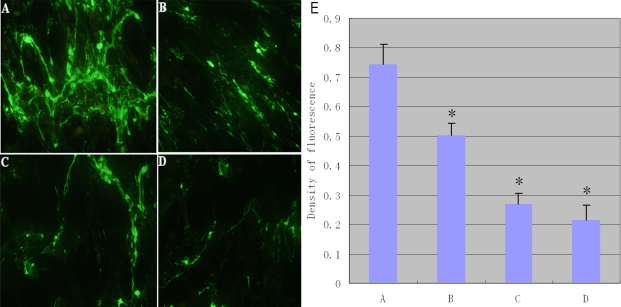
The immumofluorescence observation (100×) of fibronectin (Fn; green fibers) in rLEC treated with different doses of rapamycin. Panel **A** shows the control rLECs, while **B**, **C**, and **D** shows treatment of 0.1, 1, and 10 ng/ml rapamycin, repectively, for 24 h exposure. The staining intensities of Fn appeared greater without rapamycin incubation (**A**). The extracellular matrix is clearly reduced in the rapamycin-treated group. The histogram (**E**) summarizes the results of scanning densitometry of Fn. All differences were statistically significant, *p≤0.05.

**Figure 7:**
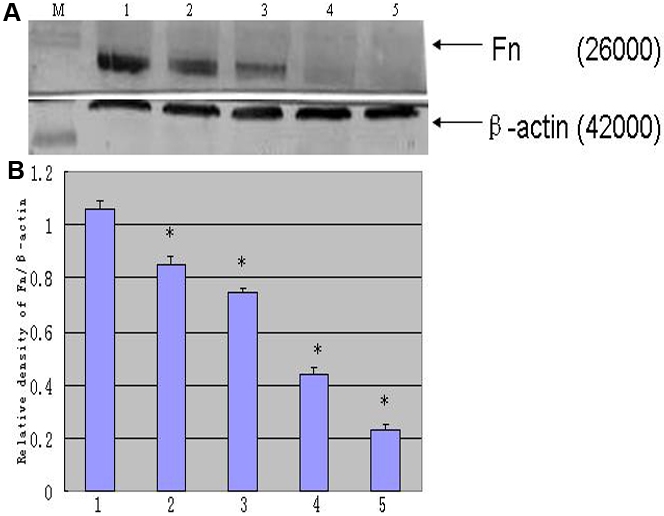
Expression of Fn in rLECs. Expression of Fn and β-actin are analyzed in panel **A**. The Fn expression is standardized by an internal control (anti-β-actin). Lanes M, 1, 2, 3, 4, and 5 are protein marker, normal control, 0.1 ng/ml for 24 h, 0.1 ng/ml for 48 h, 10 ng/ml for 24 h, 10 ng/ml rapamycin for 48 h groups, respectively. The summary results of Fn is shown in panel **B**. All differences were statistically significant (*p≤0.05).

The effect of rapamycin on the expression of intracellular Fn was evaluated in vitro (Figure 6). The results showed intense staining of fibronectin without rapamycin incubation in comparison with rapamycin incubations. As shown in Figure 7, Fn expression in rLEC was examined by western blotting. rLECs without rapamycin treatment appeared more Fn expression when compared to the rapamycin incubations. Rapamycin, decreased approximately threefold at 10 ng/ml in comparison with the control cells. Taken together, the results showed that rapamycin significantly decreased Fn expression in rLECs.

## Discussion

Cataract is the most common cause of vision impairment in the world today. It is treatable with highly effective surgery, but PCO is the most frequent complication of even successful cataract surgery. Despite several improvements in cataract surgical procedures and IOLs design, the problem of PCO is still not solved. If PCO could be prevented, patients would be more beneficial from cataract surgery. Currently available means and approaches to prevent or delay PCO are not satisfactory.

Noninvasive means of treating PCO while maintaining the integrity of the posterior capsule are being investigated. In our past study, PCO was inhibited by IOLs loaded with rapamycin in rabbit model for 24 weeks [8]. In our study, proliferation of rLECs was inhibited as low as 0.1 ng/ml rapamycin for 48 h when compared to the control cells. Rapamycin showed an inhibition of rLECs in a time- and dose-dependent manner. In our previous studies, rapamycin at dose of 1.10±0.32 μg/ml in anterior chamber postoperative 7 days could effectively inhibit PCO by PLGA regulation [8]. When using a heparin sustained drug delivery system, the benefits of a prolonged treatment period at least also have been observed by Xie et al. [10]. In that experimental, PCO occurred later with heparin sustained drug system by slow-releasing heparin into anterior chamber for 8 weeks. Because proliferation of the remaining LEC may is a continuous process starting within a few hours after cataract surgery, inhibitory proliferation of LEC with programmable release pharmacokinetics like these described by Finkelstein et al. [11] may be an effective strategy in the prevention of PCO. In our previous study, rapamycin concentration peak was observed on 24 h and continued to release till 14 days, which is long enough to inhibit LEC proliferation; consequently, it will prevent PCO formation after surgery.

Lens epithelium cellular migration also is key step in occurrence of PCO. In the present study, we examined the effect of rapamycin on rLECs for the PCO process. We found that the rLEC migration was suppressed by rapamycin treatments in a dose dependent manner. When bFGF-stimulated LECs, rapamycin even more than 0.1 ng/ml for 24 h significantly inhibited migration of rLEC when compared to the control cells (Figure 5). This dose also has been accepted as one of the most potent chemoattractants in PCO formation [12,13]. Kwon et al. [14] examined the effect of rapamycin on the migration of human dermal microvascular endothelial cells (HDMECs), which inhibited the migration of HDMECs at dose of 10 ng/ml for 6 h. Poon et al. [15] found acute administration of rapamycin had no effect on the rat aortic SMC migration, suggesting that longer exposure to rapamycin (>6 h) is required. In that study, rapamycin as low as 2 ng/ml had significant inhibitory effect on platelet-derived growth factor (PDGF)-BB homodimer-induced rat aortic SMCs migration at 48 h.

Thus, rapamycin treatment had an inhibitory effect not only on cell proliferation and migration, but also on matrix synthesis. Fn is the component of the lens capsule except for type IV collagen, heparan sulfate proteoglycans, laminin, and entactin [16-19]. Clearly one important role for capsular ECM components is to maintain the structural integrity and functional properties of the capsule. Other important roles include providing a substratum for lens cell attachment and migration [20-22]. Taliana et al. [23] assessed that the behavior of explanted lens epithelial cells on vitronectin or fibronectin substrata. On that most of the cells became elongated, spindle-shaped and were strongly reactive for filamentous α-smooth muscle actin which was typical of the well known transforming growth factor (TGF) β-induced EMT. Fn has been shown to be important in promoting the adhesion and migration of many other cells types tested under similar conditions [24,25]. Therefore, when considering strategies to slow or prevent PCO, these results highlight the need to take into account ECM molecules such as fibronectin that have the capacity to promote EMT, adhesion and migration.

Taken together, using an inhibitor of lens epithelium cell proliferation, migration and secreting fibronectin, rapamycin may have a potential inhibition of posterior capsule opacifation (PCO) and needs further study.

## References

[1] Apple DJ Solomon KD Tetz MR Assia EI Holland EY Legler UF Tsai JC Castaneda VE Hoggatt JP Kostick AM Posterior capsule opacification. Surv Ophthalmol 1992 37 73 116 1455302 10.1016/0039-6257(92)90073-3

[2] McDonnell PJ Zarbin MA Green WR Posterior capsule opacification in pseudophakic eyes. Ophthalmology 1983 90 1548 53 6677855 10.1016/s0161-6420(83)34350-5

[3] Meacock WR Spalton DJ Stanford MR Role of cytokines in the pathogenesis of posterior capsule opacification. Br J Ophthalmol 2000 84 332 6 10684849 10.1136/bjo.84.3.332 PMC1723397

[4] Saika S Ohmi S Kanagawa R Tanaka S Ohnishi Y Ooshima A Yamanaka A Lens epithelial cell outgrowth and matrix formation on intraocular lenses in rabbit eyes. J Cataract Refract Surg 1996 22 835 40 9279681 10.1016/s0886-3350(96)80171-4

[5] Moses JW Leon MB Popma JJ Fitzgerald PJ Holmes DR O'Shaughnessy C Caputo RP Kereiakes DJ Williams DO Teirstein PS Jaeger JL Kuntz RE SIRIUS Investigators. Sirolimus-eluting stents versus standard stents in patients with stenosis in a native coronary artery. N Engl J Med 2003 349 1315 23 14523139 10.1056/NEJMoa035071

[6] Suzuki T Kopia G Hayashi S Bailey LR Llanos G Wilensky R Klugherz BD Papandreou G Narayan P Leon MB Yeung AC Tio F Tsao PS Falotico R Carter AJ Stent-based delivery of sirolimus reduces neointimal formation in a porcine coronary model. Circulation 2001 104 1188 93 11535578 10.1161/hc3601.093987

[7] Poon M Marx SO Gallo R Badimon JJ Taubman MB Marks AR Rapamycin inhibits vascular smooth muscle cell migration. J Clin Invest 1996 98 2277 83 8941644 10.1172/JCI119038 PMC507677

[8] Liu H Wu L Fu S Hou Y Liu P Cui H Liu J Xing L Zhang X Polylactide-glycoli acid and rapamycin coating intraocular lens prevent posterior capsular opacification in rabbit eyes. Graefes Arch Clin Exp Ophthalmol 2009 247 801 7 19066932 10.1007/s00417-008-1007-0

[9] Mosmann T Rapid colorimetric assay for cellular growth and survival: application to proliferation and cytotoxicity assays. J Immunol Methods 1983 65 55 63 6606682 10.1016/0022-1759(83)90303-4

[10] Xie L Sun J Yao Z Heparin drug delivery system for prevention of posterior capsular opacification in rabbit eyes. Graefes Arch Clin Exp Ophthalmol 2003 241 309 13 12719992 10.1007/s00417-003-0645-5

[11] Finkelstein A McClean D Kar S Takizawa K Varghese K Baek N Park K Fishbein MC Makkar R Litvack F Eigler NL Local drug delivery via a coronary stent with programmable release pharmacokinetics. Circulation 2003 107 777 84 12578884 10.1161/01.cir.0000050367.65079.71

[12] Ibaraki N Lin LR Reddy VN Effects of growth factors on proliferation and differentiation in human lens epithelial cells in early subculture. Invest Ophthalmol Vis Sci 1995 36 2304 12 7558725

[13] Wunderlich K Knorr M Effect of platelet-derived growth factor PDGF on replication of cultivated bovine lens epithelial cells. Ophthalmologe 1994 91 98 102 8173260

[14] Kwon YS Kim JC Inhibition of corneal neovascularization by rapamycin. Exp Mol Med 2006 38 173 9 16672771 10.1038/emm.2006.21

[15] Poon M Marx SO Gallo R Badimon JJ Taubman MB Marks AR Rapamycin inhibits vascular smooth muscle cell migration. J Clin Invest 1996 98 2277 83 8941644 10.1172/JCI119038 PMC507677

[16] Parmigiani C McAvoy J Localisation of laminin and fibronectin during rat lens morphogenesis. Differentiation 1984 28 53 61 6394411 10.1111/j.1432-0436.1984.tb00266.x

[17] Cammarata PR Cantu-Crouch D Oakford L Morrill A Macromolecular organization of bovine lens capsule. Tissue Cell 1986 18 83 97 3515629 10.1016/0040-8166(86)90009-1

[18] Marshall GE Konstas AG Bechrakis NE Lee WR An immunoelectron microscope study of the aged human lens capsule. Exp Eye Res 1992 54 393 401 1521568 10.1016/0014-4835(92)90051-s

[19] Majo F Montard M Delbosc B Kantelip B Immunolabelling of collagen types I, III and IV, laminin and fibronectin in the human lens capsule. J Fr Ophtalmol 1997 20 664 70 9587577

[20] Zelenka PS Regulation of cell adhesion and migration in lens development. Int J Dev Biol 2004 48 857 65 15558477 10.1387/ijdb.041871pz

[21] Cammarata PR Spiro RG Identification of noncollagenous components of calf lens capsule: evaluation of their adhesion-promoting activity. J Cell Physiol 1985 125 393 402 3905828 10.1002/jcp.1041250306

[22] Cammarata PR Spiro RG Lens epithelial cell adhesion to lens capsule: a model system for cell-basement membrane model system for cell-basement membrane interaction. J Cell Physiol 1982 113 273 80 7174730 10.1002/jcp.1041130215

[23] Taliana L Evans MD Ang S McAvoy JW Vitronectin is present in epithelial cells of the intact lens and promotes epithelial mesenchymal transition in lens epithelial explants. Mol Vis 2006 12 1233 42 17110906

[24] McCarthy JB Hagen ST Furcht LT Human fibronectin contains distinct adhesion- and motility-promoting domains for metastatic melanoma cells. J Cell Biol 1986 102 179 88 3941152 10.1083/jcb.102.1.179 PMC2114050

[25] Herbst TJ McCarthy JB Tsilibary EC Furcht LT Differential effects of laminin, intact type IV collagen, and specific domains of type IV collagen on endothelial cell adhesion and migration. J Cell Biol 1988 106 1365 73 3360855 10.1083/jcb.106.4.1365 PMC2115013

